# Efficient Adsorption of Tebuconazole in Aqueous Solution by Calcium Modified Water Hyacinth-Based Biochar: Adsorption Kinetics, Mechanism, and Feasibility

**DOI:** 10.3390/molecules28083478

**Published:** 2023-04-14

**Authors:** Yucan Liu, Zhonglu Gao, Xianguo Ji, Ying Wang, Yan Zhang, Hongwei Sun, Wei Li, Lide Wang, Jinming Duan

**Affiliations:** 1 School of Civil Engineering, Yantai University, Yantai 264005, China; 2 School of Environmental and Materials Engineering, Yantai University, Yantai 264005, China; 3 Key Laboratory of Northwest Water Resources, Environment and Ecology, Ministry of Education, Xi’an University of Architecture and Technology, Xi’an 710055, China; 4 Ningxia Branch of China Design Group Co., Ltd., Yinchuan 750001, China; 5 Centre for Water Management and Reuse, University of South Australia, Mawson Lakes Campus, Adelaide, SA 5095, Australia

**Keywords:** water hyacinth, biochar, calcium modification, adsorption, tebuconazole

## Abstract

The application of fungicides (such as tebuconazole) can impose harmful impacts on the ecosystem and humans. In this study, a new calcium modified water hyacinth-based biochar (WHCBC) was prepared and its effectiveness for removing tebuconazole (TE) via adsorption from water was tested. The results showed that Ca was loaded chemically (CaC_2_O_4_) onto the surface of WHCBC. The adsorption capacity of the modified biochar increased by 2.5 times in comparison to that of the unmodified water hyacinth biochar. The enhanced adsorption was attributed to the improved chemical adsorption capacity of the biochar through calcium modification. The adsorption data were better fitted to the pseudo-second-order kinetics and the Langmuir isotherm model, indicating that the adsorption process was dominated by monolayer adsorption. It was found that liquid film diffusion was the main rate-limiting step in the adsorption process. The maximum adsorption capacity of WHCBC was 40.5 mg/g for TE. The results indicate that the absorption mechanisms involved surface complexation, hydrogen bonding, and π–π interactions. The inhibitory rate of Cu^2+^ and Ca^2+^ on the adsorption of TE by WHCBC were at 4.05–22.8%. In contrast, the presence of other coexisting cations (Cr^6+^, K^+^, Mg^2+^, Pb^2+^), as well as natural organic matter (humic acid), could promote the adsorption of TE by 4.45–20.9%. In addition, the regeneration rate of WHCBC was able to reach up to 83.3% after five regeneration cycles by desorption stirring with 0.2 mol/L HCl (*t* = 360 min). The results suggest that WHCBC has a potential in application for removing TE from water.

## 1. Introduction

Pesticides have been widely used in modern agricultural industries for promoting production. Tebuconazole (TE), a representative product of triazole fungicides [1], can inhibit the biosynthesis of ergosterol from fungi and has been used for the prevention and control of fungal hazards in crops. Due to its high efficiency and broad bactericidal spectrum as well as long validity, TE has been widely used for over 60 crops in more than 50 countries around the world [2]. However, the application of TE may result in an accumulation in the natural environment due to its high stability and low biodegradability. Previous studies have shown that its detection rate and concentration in the environment and wastewater treatment plants were relatively high. For example, Bollmann et al. [3] reported that TE concentration in wastewater ranged from ng/L to μg/L, and the mass load of TE in the influent was 6 mg/h in dry weather and up to 3–68 mg/h in wet weather. Studies have shown that TE in drinking water sources may pose potential carcinogenic risks and toxicological impacts to humans, such as thyroid endocrine disorders, developmental toxicity, and embryo toxicity [4,5,6]. However, conventional water treatment processes are ineffective in removing TE from water [7]. Therefore, the removal of TE from drinking water may become essential under certain source water conditions for a safe drinking water supply.

The current methods for removing micro-organic pollutants from water include biological treatment [8], photocatalytic oxidation [9,10], electrochemical degradation [11], and adsorption [12]. However, most of the existing methods have certain limitations, such as insufficient flexibility and high costs of application processing. For example, biological treatment technologies require stable environmental factors (such as temperature and pH) and skilled manpower to operate and maintain [13]. Although advanced oxidation processes (AOPs) are effective in the removal of organic pollutants from water [14,15], the high energy consumption and catalyst consumption in practical water treatment projects make this method much less applicable [16]. In comparison, adsorption may be potentially more advantageous for removing the persistent organic pollutants (POPs) from water because of its high efficiency, low-cost, and simple operation [17]. Presently, the adsorption materials used to remove TE from water include composite materials [18], organic frameworks [19], and activated carbon [12]. However, the preparation methods of most adsorption materials are complex and the costs are difficult to manage, thus limiting their use as adsorbents. Biochar is a carbon-rich material with multifunctional uses, such as adsorption and catalysis, which is caused by the pyrolysis process of the biomass [20,21,22]. Compared with activated carbon [23], the raw materials for preparing biochar exist widely in the natural environment, and secondary pollution barely exists during the preparation process. Moreover, waste biomass, such as agricultural wastes, crop residues, and animal wastes, can all be used as raw materials for biochar production. The application of biochar adsorbents can not only facilitate the reuse of waste resources but also has the advantage of providing renewable resources and mitigating global climate change [24].

The water hyacinth (Eichhornia crassipes), one of the world’s 100 most aggressive invasive species, as listed by the International Union for Conservation of Nature (IUCN), has a strong reproductive capacity in aquatic systems [25]. The decaying roots and leaves of the water hyacinth can lead to the deterioration of water quality and affect the growth and survival of other aquatic plants and animals, potentially causing great harm to aquatic environments if not treated in time. The water hyacinth can be used as a raw material for biochar production, as its waste can be harvested and utilized. Previous studies have found that there are many hydroxyl groups and other functional groups on the cellulose skeleton of the water hyacinth, which makes the water hyacinth more conducive to the preparation of efficient biochar [26]. Currently, the water hyacinth as a raw material for the preparation of biochar has been widely studied for the removal of organic matter and harmful inorganic substances in water and wastewater. For example, Viswanathan et al. [27] studied the adsorption potential of water hyacinth biochar for organic matter such as methylene blue and crystalline violet in aqueous media with a maximum removal rate of 96.2%. Ramirez-Muñoz et al. [28] prepared water hyacinth biochar to adsorb phosphorus in aqueous solutions with a maximum adsorption capacity of 21.21 mg/g. These studies have confirmed that water hyacinth biochar can be used for pollutant removal from water and has excellent engineering application potential. However, the application of unmodified biochar is likely limited by its own insufficient specific surface area, small pore size, poor anti-interference ability, and its adsorption capacity for removing pollutants [29]. It appears that the appropriate modification of the raw biochar may significantly improve its adsorption performance for pollutant removal from water.

Metal modification is one of the commonly used modification methods for biochar preparation. Biochar modified by magnesium, iron, and calcium has been proven to be effective in improving the adsorption performance of biochar. For example, Lu et al. [30] investigated the performance of magnetic biochar composites for the removal of azole fungicides, and the maximum adsorption capacity of epoxiconazole and flusilazole was 86.11 mg/g and 89.87 mg/g, respectively. Wang et al. [31] prepared FeCl_3_-modified biochar with corn straw as a raw material, which was able to effectively remove carbendazim from wastewater (adsorption capacity of 108.1 mg/g). Therefore, it is feasible to use certain metal salts to modify water hyacinth-based biochar (WHBC) in order to enhance the adsorption capacity of fungicides from water. However, most metal modifications also have disadvantages—the leaching of metal modifiers that may occur in biochar can cause additional heavy metal pollution to the water quality and environment [32]. Among the metal ion modifications, calcium is an environmentally friendly substance, which has attracted more and more attention because of its ecological harmlessness and relevant properties [33]. In addition, previous studies have shown that calcium modification can effectively increase the adsorption capacity of biochar. For example, Zhuo et al. [34] reported that modified corn stover biochar was prepared using calcium chloride as a modifier. The adsorption capacities of biochar for phosphate and tetracycline were increased from 0 mg/g and 7.1 mg/g for the unmodified biochar to 25.8 mg/g and 17.7 mg/g after modification, respectively. Wallace, Anna, et al. [35] investigated the removal of fluoride from water using modified dairy manure-based biochar with calcium chloride as a modifier and showed a 3.82–8.86 times higher removal of fluoride from water through the modified biochar than via the original manure-based biochar. Calcium ion modification was able to effectively improve the adsorption capacity of biochar without the concerns over secondary pollution to the water quality. Therefore, the use of calcium modified biochar to remove fungicides, such as TE, in water may be feasible and promising. To our knowledge, there are few studies on the adsorption removal of TE by biochar and the mechanism of adsorption is not very clear. In addition, calcium modified water hyacinth-based biochar (WHCBC) has not been studied. 

In this study, calcium chloride was used as a modifier to prepare WHCBC. The adsorption performance of the modified biochar for TE was investigated accordingly. This study aimed to (a) study the effect of calcium modification on the surface morphology and microstructure of WHCBC; (b) investigate the effects of reaction conditions (carbon dosage, initial TE concentration, initial solution pH, and solution temperature) on the adsorption of TE by WHCBC; (c) investigate the adsorption kinetics and adsorption isotherms of TE by WHCBC; (d) elucidate the adsorption mechanism of WHCBC for TE; and (e) evaluate the stability, safety, and reusability of WHCBC. 

## 2. Results and Discussion

### 2.1. Characterization of Biochar

#### 2.1.1. SEM–EDS Analysis 

**Figure 1:**
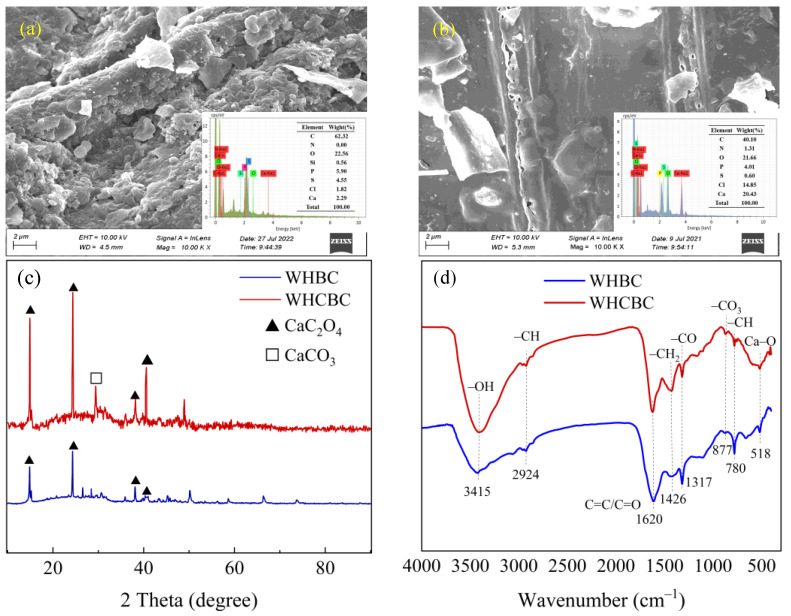
Characterization of WHBC and WHCBC: (**a**) SEM–EDS and element content of WHBC; (**b**) SEM–EDS and element content of WHCBC; (**c**) XRD pattern comparison of WHBC and WHCBC; (**d**) Spectral comparison of FTIR of WHBC and WHCBC.

The SEM images indicate that the surface structure of WHBC was rough and disorganized (Figure 1a). Due to the irregular fragments deposited on the surface of biochar, its pore structure could not be identified. In contrast, the surface of WHCBC modified by Ca became flatter and smoother, and the pores of different sizes could be observed. The EDS analysis showed that the content of Ca increased significantly as one of the major elements on WHCBC compared with WHBC, which confirmed the successful loading of Ca on biochar (Figure 1b). According to Figure 1b, the Ca loaded on the surface of WHCBC was in the form of large particles, which caused a noticeable change in the surface morphology of biochar. This was similar to the findings of a previous study on modified biochar with Ca [33].

#### 2.1.2. Brunauer–Emmett–Teller Analysis

Figure S3 shows the Brunauer–Emmett–Teller (BET) adsorption/desorption isotherms and pore size distribution of WHBC and WHCBC. The results indicate that the specific surface area and the pore size of WHCBC increased after modification with calcium. The adsorption/desorption curve of WHCBC had an H3 hysteresis loop. The curve type was a typical type IV isotherm, according to the International Union of Pure and Applied Chemistry classification (Figure S3a), which was of the characteristics for mesoporous materials [36]. In addition, the pore volume and average pore size of WHCBC were higher than those of WHBC. This may be due to the collapse of the pore wall caused by the calcium modification of the biochar [37], which increased the pore size and thus significantly increased the pore volume of the mesopores after modification (Table 1). It is well known that the larger the specific surface area and pore volume of the biochar, the higher the physical adsorption capacity of the biochar. Therefore, WHCBC is able to provide more adsorption sites for TE in the adsorption process (see later sections).

#### 2.1.3. X-ray Diffraction Analysis

The comparative analysis of WHBC and WHCBC using X-ray Diffraction (XRD) provides evidence for the successful modification of WHCBC (Figure 1c). Compared with WHBC, the characteristic peak of calcium oxalate (CaC_2_O_4_, 2θ = 14.86°, 24.32°, 38.10°, and 40.56°) and calcium carbonate (CaCO_3_, 2θ = 29.4°) were significantly enhanced after modification, indicating that Ca ions were chemically bound on the biochar. In addition, the calcium on the biochar was dominated by calcium oxalate. Calcium carbonate was also detected in the modified biochar, which may be due to the decomposition of calcium oxalate at high pyrolysis temperatures [38]. 

#### 2.1.4. Fourier Transform Infrared Spectral Analysis

The changes of chemical functional groups on biochar before (WHBC) and after (WHCBC) modification were revealed by Fourier transform infrared (FTIR) spectra (Figure 1d). The –OH telescopic peak appeared at 3415 cm^−1^, while the peak at 2924 cm^−1^ could be attributed to the C–H asymmetric vibration of the aliphatic group. The peak at 1620 cm^−1^ was attributed to the stretching vibration of the C=C, indicating the presence of an aromatic or graphite structure. It is known that organic pollutants were easily adsorbed via π–π interactions [39]. The C–O stretching vibration appeared at 1317 cm^−1^, while the peaks at 1426 cm^−1^ and 780 cm^−1^ were attributed to the stretching vibration of –CH_2_ and aromatic –CH out-of-plane bending vibrations [40]. The peak at 877 cm^−1^ was attributed to the stretching vibration of –CO_3_. After Ca modification, a clear peak alteration at 518 cm^−1^ was detected to be related to Ca–O vibration, which proved that Ca ions are chemically bound to the biochar [41]. These results consolidate the observations made via XRD analysis.

### 2.2. Adsorption Capacity

#### 2.2.1. Effect of Biochar Dosages and Initial TE Concentration

**Figure 2:**
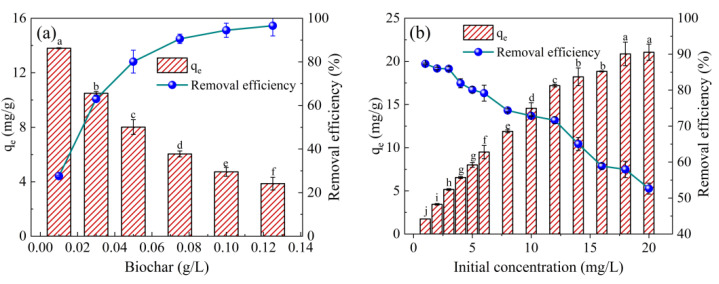

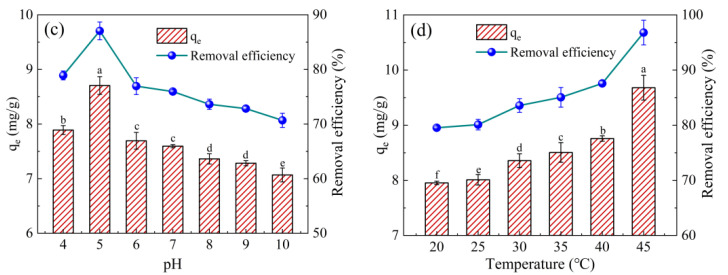
Effect of experimental conditions on adsorption of TE by WHCBC: (**a**) WHCBC dosage; (**b**) initial TE concentration; (**c**) solution pH; (**d**) solution temperature (*C*_0_ = 5 mg/L, *m* = 0.05 g, *t* = 0–240 min, *T* = 25 °C). Different letters indicate significant differences between different adsorption conditions at the 0.05 level, tested via one-way ANOVA.

The dosage of biochar affects the removal rate of pollutants in an aqueous solution because the increase in the amount of adsorbent increases the surface area and binding sites of adsorbents. The results show that the WHCBC adsorption capacity was 13.80, 10.50, and 8.01 mg/g at the WHCBC dosages of 0.10, 0.30, and 0.50 g/L, respectively, while the TE removal rates were 27.6%, 63.0%, and 80.0%, respectively (Figure 2a). As can be seen from Figure 2a, the binding rate of the WHCBC surface binding sites was high at lower dosages and WHCBC exhibited excellent adsorption properties. The adsorption capacity of WHCBC decreased with increasing dosages, which was attributed to a decrease in the ratio of TE molecules to adsorption sites, resulting in a decrease in the unit adsorption capacity. When the amount of biochar was greater than 0.5 g/L, the adsorption capacity of WHCBC tended to stabilize, which was mainly because the number of TE molecules was relatively small and there were excess available adsorption sites during the final adsorption process. Similar results have also been reported in other studies on biochar [42]. 

Initial TE concentration was also an important factor for TE adsorption from aqueous solution by WHCBC. The adsorption capacities of WHCBC were 1.75, 8.01, 13.17, and 21.09 mg/g at initial TE concentrations of 1, 5, 10, and 20 mg/L, respectively. The TE removal rates were 87.30%, 80.10%, 72.85%, and 52.64%, respectively (Figure 2b). The results showed that the adsorption capacity of WHCBC increased with the increase in initial TE concentration in the aqueous solutions. This was because the driving force for the diffusion of the adsorbate from the bulk liquid phase to the adsorbent surface increased with the increase in TE concentration in the aqueous solution, resulting in a large number of adsorbate molecules to be adsorbed on the adsorbent surface. This allowed the efficient utilization of the adsorption sites of WHCBC and increased the adsorption capacity of WHCBC significantly (*p* < 0.05). However, the adsorption sites of WHCBC were gradually saturated with the further increase in TE concentration in the aqueous solution, so the excessive concentration of TE led to a reduction in its removal rate. Similar results were obtained in the study of dye wastewater treatment by Li et al. [43]. In the present study, it was found that TE removal rate started to decrease when the initial TE concentration was greater than 5 mg/L. In order to investigate the mechanism of TE adsorption by WHCBC, and similarly to previous studies on related fungicides [44], an initial TE concentration of 5 mg/L was selected for subsequent experiments. 

#### 2.2.2. Effect of Initial pH and Temperature

The solution pH affects the surface charge and ionization degree of the adsorbent, thus affecting the adsorption process [43]. The isoelectric point (iep) of WHCBC was observed at approximately pH = 4.85 in the present study (see Figure S4). The adsorption of TE on WHCBC first increased and then decreased between pH 4–10; the maximum adsorption capacity of WHCBC was 8.71 mg/g at pH 5 (Figure 2c). The pK_a_ of TE was reported to be at 5.0 ± 0.1 [45], close to the pH_iep_ of WHCBC. Therefore, when pH < pH_iep_, the surface of WHCBC and TE was positively charged, which led to mutual repulsion of WHCBC and TE, hindering TE adsorption on the surface of WHCBC and thus reducing the adsorption capacity of TE on WHCBC. In addition, under low solution pH conditions, H^+^ may compete with TE for adsorption sites on WHCBC, reducing the adsorption capacity of TE with the reactive groups on the WHCBC surface [43]. On the contrary, the surfaces of WHCBC and TE were negatively charged when the solution pH was greater than the pK_a_ of TE, so electrostatic repulsion prevented the adsorption of TE by WHCBC. Electrostatic repulsion increases with the increase in solution pH. This may explain why the adsorption capacity of WHCBC decreased from 8.71 mg/g at a pH of 5 to 7.07 mg/g at a pH of 10. When the solution’s pH was around 5, the surface of WHCBC and TE were both electrically neutral and had no electrostatic interaction or repulsion, so the maximum adsorption capacity of WHCBC was obtained (8.71 mg/g). In the present study, the electrostatic effect had a significant influence on the adsorption capacity of WHCBC (*p* < 0.05), although other mechanisms might be also involved. At the same time, the results also indicate that WHCBC was able to maintain a relatively stable and large adsorption capacity in a wide solution pH range.

Temperature is another factor that affects adsorption at the solid–liquid interface. As shown in Figure 2d, the adsorption capacity of TE on the WHCBC from 7.95 mg/g at 20 °C increased to 9.68 mg/g at 45 °C, and the TE removal rate from 79.53% at 20 °C increased to 96.79% at 45 °C (*p* < 0.05). The results indicate that the adsorption capacity (*q*_e_) of WHCBC was positively correlated with the solution temperature. The adsorption capacity of TE on the WHCBC increases with the increase in solution temperature. It may be speculated that the thermal motion of TE molecules was more intense and the driving force of diffusion through the adsorbing particle’s boundary layer was enhanced with the increase in temperature [46]. This promoted the adsorption of TE by WHCBC.

### 2.3. Adsorption Kinetics

**Figure 3:**
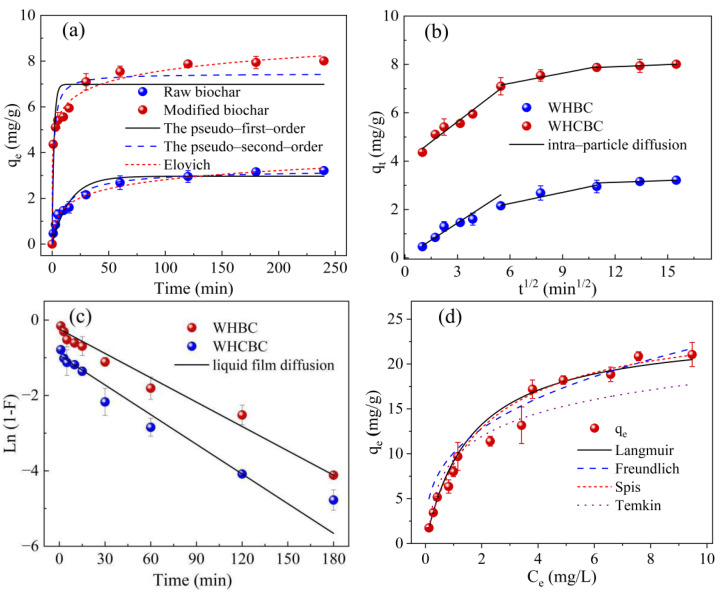

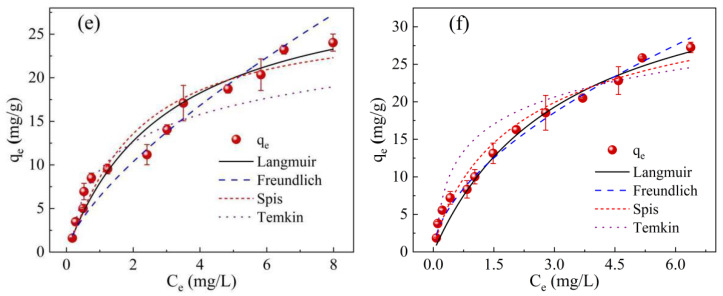
The adsorption kinetics and isotherms of TE on the two kinds of biochar: (**a**–**c**) adsorption kinetics of WHBC and WHCBC at 5 mg/L TE in water; and (**d**–**f**) are the adsorption isotherms of WHCBC at temperatures of 25 °C, 35 °C, and 45 °C, respectively.

The kinetics of TE adsorption process were studied, and the properties of unmodified biochar (WHBC) and modified biochar (WHCBC) were compared (Figure 3a). The results indicated that TE was rapidly adsorbed by the biochar in the first 30 min. This was due to the high concentration of TE in the solution and the large number of adsorption sites provided by the biochar surface at the initial stage of the adsorption. The adsorption rate of TE on the biochars gradually decreased at 30–120 min, and this was because the concentration of TE in the aqueous solution decreased gradually with increasing adsorption contact time. At the later stage of adsorption (>120 min), the adsorption sites on the surface of was WHCBC gradually saturated, and TE could diffuse inside the biochar particles and occupy the adsorption sites on the inner surface of the biochar adsorbent, thus reaching the adsorption equilibrium at 240 min.

The adsorption capacity of TE by biochar was increased from 3.21 mg/g on WHBC to 8.01 mg/g on WHCBC (Figure 3a). The adsorption capacity of WHCBC for TE was 2.5 times that of WHBC. The adsorption rate constant *K* of WHCBC was much higher than that of WHBC (Table 2), indicating that WHCBC had a much higher removal efficiency for TE. 

To further analyze the adsorption kinetics of TE by the biochar, five kinetic models were used to fit the adsorption data. The relevant parameters are shown in Table 2. Compared with the pseudo-first-order kinetics (*R*^2^ = 0.805), the pseudo-second-order kinetic model (*R*^2^ = 0.911) and the Elovich model (*R*^2^ = 0.989) are able to better fit the adsorption process. Both models were suitable for describing the chemically dominated dynamic adsorption process and indicated that the adsorbate and adsorbent involved electron transfer and sharing during the adsorption process [47]. The liquid film diffusion model and the intra-particle diffusion model could explore the diffusion mechanism and the possible limiting steps of the adsorption rate. The fitting results show that the curves of WHBC and WHCBC exhibited three linear segments in the intra-particle diffusion, indicating that the diffusion process consists of three stages (boundary layer diffusion, intra-particle diffusion, and internal surface adsorption). As shown in Figure 3c, the higher adsorption rate in the first stage was due to the large number of adsorption sites on the adsorbent during the initial stage of adsorption. While the adsorption sites were gradually occupied as the adsorption process proceeded, the adsorption rate in the second and third stages gradually decreased until the adsorption equilibrium was reached. It is worth noting that the non-zero intercept (*C*_i_) of each stage indicated that intra-particle diffusion was not the sole rate-limiting step in the adsorption process. Particularly for WHCBC, the large intercept (*C*_i_) indicated that intraparticle diffusion was not the main control step, in contrast to the high *R*^2^ value (1.00) of the liquid film diffusion model. This could confirm the important involvement of liquid film diffusion in the adsorption process. That is, it is the main rate-limiting step in the adsorption process [48]. In addition, the larger intercept in the intra-particle diffusion model indicates that the contribution of surface adsorption to the adsorption process was greater, while WHCBC has a lower contribution of pore adsorption; thus, intrapore absorption was not the main adsorption mechanism for WHCBC.

### 2.4. Adsorption Isotherms

As shown in Table 3, the correlation coefficient fitted by the Langmuir model (*R*^2^ = 0.989) is better than that of the Freundlich model (*R*^2^ = 0.980). This may indicate that the adsorption of TE by WHCBC is mainly monolayer adsorption [49]. 

Meanwhile, when the solution temperature was at 25 °C, 35 °C, and 45 °C, the maximum adsorption capacity of WHCBC fitted by the Langmuir model was 24.0 mg/g, 32.2 mg/g, and 40.5 mg/g, respectively. WHCBC had a better adsorption capacity for TE than previously reported iron modified sludge-based biochar (11.3 mg/g) [50]. The Freundlich model parameter 1/*n* was used to define the favorability of the adsorption system, with 1/*n* ranging from 0 to 1 for good, and indicated the adsorption was predominantly chemisorption [51]. Based on the value of 1/*n*, WHCBC had a favorable adsorption process, indicating that the excellent chemisorption performance of WHCBC was achieved. Furthermore, the high *R*^2^ values (0.980 < *R*^2^ < 0.989) of the WHCBC adsorption curve fitted using the Sips model demonstrated that the adsorption of TE on WHCBC was relatively complicated and could not be explained by the use of only one model. The adsorption process might not only be a monolayer adsorption of uniform sites but might also contain the diversity of adsorption sites on the WHCBC surface [52]. The high correlation coefficient fitted by the Temkin isotherm model (0.942 < *R*^2^ < 0.973) also illustrated this point [53]. The above results demonstrate that WHCBC is an effective adsorbent for TE removal from water.

### 2.5. Adsorption Mechanism

**Figure 4:**
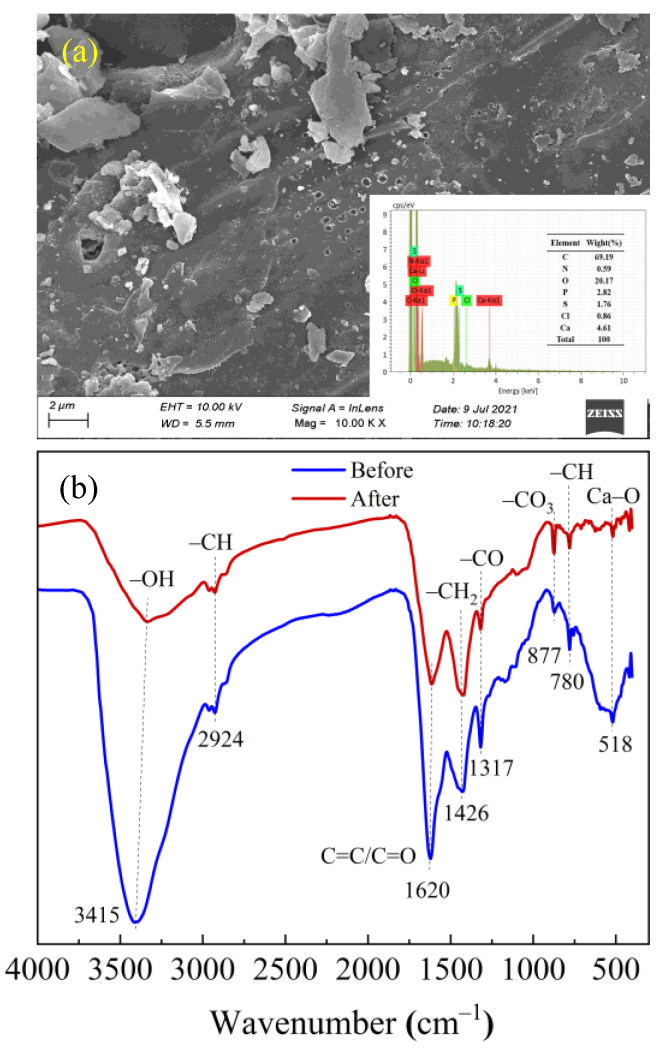
Characterization of WHCBC after adsorption: (**a**) SEM–EDS; (**b**) FTIR spectrum.

To elucidate the adsorption mechanism of TE on WHCBC, a series of characterizations of WHCBC before and after adsorption tests were performed. SEM images showed that particles of different sizes were attached to the surface and pores of WHCBC after adsorption (Figure 4a). This analysis was further confirmed by EDS results. The C content of WHCBC increased from 40.10% before adsorption to 69.19% after adsorption, while the Ca content of WHCBC decreased from 20.43% before adsorption to 4.61% after adsorption. This may also indicate that TE was adsorbed by WHCBC, that the Ca load on WHCBC participated in the adsorption reaction, and that surface complexation might have occurred [54].

The changes of functional groups of WHCBC before and after adsorption of TE was observed through FTIR (Figure 4b). The stretching vibration band of 3415 cm^–1^ was greatly weakened after adsorption, indicating that –OH on the surface of WHCBC was participating in the adsorption reaction. This indicated that hydrogen bonding was one of the main adsorption mechanisms of TE by WHCBC [55]. The decrease in the intensity of the C=C peak at 1620 cm^–1^ indicated that π–π interaction was involved in TE adsorption [34]. Previous studies proved that carbon materials had a strong potential to accept electrons and could act as π-electron acceptors [56]. In addition, –OH in the TE could provide electrons and act as π-electron donors [18]. Therefore, the π–π electron donor–acceptor interaction between WHCBC and TE was one of the main contributions to the improvement of the adsorption capacity. This might also be the reason why the stretching vibration of the –CH_2_ peak represented by 1416 cm^–1^ was significantly enhanced compared with that before adsorption [51]. The change in the –CO_3_ peak at 877 cm^–1^ was due to the effect of adsorption on oxygen-containing functional groups, while the change of the peaks located at 1317 cm^–1^ and 780 cm^–1^ were attributed to the vibration of –CO and –CH peaks caused by π–π interaction. The obvious decrease in the peak at 518 cm^–1^ indicated that Ca–O might be involved in the complexation reaction. This was also indicated by SEM–EDS, indicating that surface complexation was one of the main adsorption mechanisms of TE by WHCBC [57].

**Figure 5:**
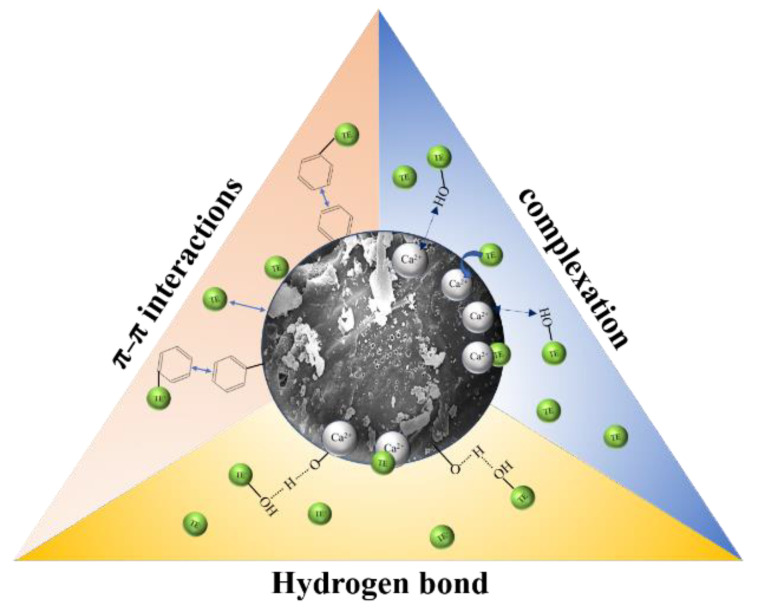
Schematic diagram of adsorption mechanism of TE by WHCBC.

To summarize, the main adsorption mechanisms of TE by WHCBC included surface complexation, hydrogen bonding, and π–π interaction (Figure 5).

### 2.6. Effect of Coexisting Substances, Leaching, and Renewability

**Figure 6:**
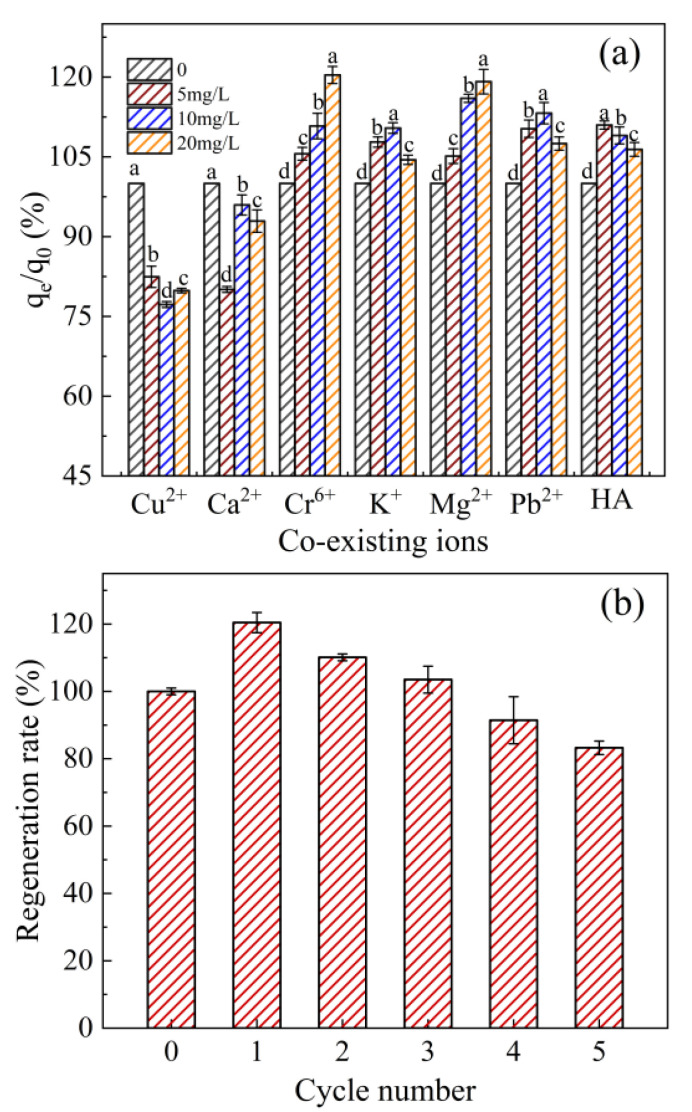
Effects of coexisting substances and reusability of WHCBC: (**a**) effects of coexisting ions (Cu^2+^, Ca^2+^, Cr^6+^, K^+^, Mg^2+^, Pb^2+^) and HA on TE removal; (**b**) regeneration rate of WHCBC. Different letters indicate significant differences between the different concentrations of coexisting substances at the 0.05 level, tested by one-way ANOVA.

The practicability of WHCBC could be evaluated to some extent by investigating the impact of coexisting substances on the adsorption capacity of WHCBC. Based on the consideration of typical coexisting substances in natural water bodies and with reference to the literature [51], six metal ions (Cu^2+^, Ca^2+^, Cr^6+^, K^+^, Mg^2+^, Pb^2+^) and HA were selected in this study to investigate their impact on the removal efficiency of TE. The inhibition effects of Cu^2+^ and Ca^2+^ on the adsorption of TE (4.05–22.8%) in water by WHCBC were observed (Figure 6a). This can be attributed to their competition for limited adsorption sites on biochar [58]. However, Cr^6+^ and Mg^2+^ significantly promoted the adsorption of TE (5.14–20.9%) by WHCBC (*p* < 0.05). This might be ascribed to the formed modified biochar–TE–Cr^6+^/Mg^2+^ or modified biochar–Cr^6+^/Mg^2+^–TE complex. Similar results have been reported by Yao et al. [59] in the removal of tetracycline and copper in aqueous solutions by modified wood biochar. The low concentration of K^+^ and Pb^2+^ (<0 mg/L) promoted the adsorption of TE (7.80–13.2%) by WHCBC (*p* < 0.05), while a high concentration of K^+^ and Pb^2+^ (> 0 mg/L) resulted in a decrease in TE adsorption (4.45–7.49%) on WHCBC. When HA concentration was 0, 5, 10, and 20 mg/L, the *q*_e_/*q*_0_ of WHCBC were 100%, 111%, 109%, and 106%, respectively. These results indicate that the low concentration (<5 mg/L) of HA played a facilitating role in the adsorption of TE from water by WHCBC (*p* < 0.05). This may be because HA itself was also an adsorbent and had a certain adsorption effect on TE. With the increase in HA concentration (>5 mg/L), the surface of WHCBC was covered by HA, leading to the blockage of WHCBC pores and the decrease in effective adsorption sites [60]. In the present study, the TE adsorption inhibition of WHCBC by Cu^2+^ and Ca^2+^ ranged from 4.05% to 22.8%. On the other hand, the presence of other coexisting cations (Cr^6+^, K^+^, Mg^2+^, Pb^2+^), as well as the natural organic matter (humic acid), was able to promote TE adsorption (4.45–20.9%). Compared to previous studies by Nguyen et al. [61] (the inhibition rate of coexisting ions > 50%) and Cao et al. [48] (the inhibition rate of coexisting ions > 80%), WHCBC had better environmental adaptability and resistance to ionic interferences.

In order to explore the safety of WHCBC in practical applications, the leaching concentrations of calcium were examined at pH 3, 5, 7, 9, and 11 (Figure S5). The results show that the leaching concentrations of calcium (0.00746–0.0321 mg/L) decreased with the increase in the pH value of the solution. The leaching experiments have shown that the calcium leaching concentration of WHCBC in water is much less than the limit values of calcium concentration in drinking water, as specified by the Standards for Drinking Water Quality of China (GB5749-2022) (hardness not greater than 450 mg/L CaCO_3_). In addition, the concentrations of other heavy metals (Fe, Cr, Cd, Pb, and As) were lower than the detection limit. This shows that WHCBC is chemically stable and environmentally safe in a wide pH range.

Regeneration characteristics are not only some of the most important indexes for evaluating the adsorption potential of adsorbents but also an important factor for investigating their application value in actual industrial water treatment systems. Therefore, an investigation into regeneration and reusability is of great significance to the practical benefits in application. In the present study, WHCBC was desorbed in 0.2 mol/L HCl with stirring after adsorbing TE and then re-adsorbed TE under the same conditions to form an adsorption–desorption cycle. The regeneration rate of WHCBC was greater than 100% after the first three adsorption–desorption cycles and even reached up to 120.43% in the first cycle (Figure 6b). The results indicate that the acid washing not only effectively washed off the adsorbate TE from WHCBC but also made the surface of WHCBC rougher, which greatly increased the surface area of WHCBC [62]. In addition, studies have shown that the pore size of the biochar surface was expanded and that more acidic functional groups appeared after acid regeneration [63]. The regeneration still desorbed most of TE in the fifth cycle, retained excellent adsorption capacity, and the adsorption rate still reached a satisfactory level (83.25%). The regeneration effect of WHCBC is better than that of regeneration rate (44.5%) after four regenerations of the Fe–N-co-modified biochar prepared by Li et al. [52], and the regeneration rate (67.1%) after five regenerations of ZnO-modified biochar prepared by Yu et al. [64]. Overall, the experiment demonstrated that WHCBC is a stable adsorbent with good reusability and cost-effectiveness.

## 3. Materials and Methods

### 3.1. Reagents and Materials

TE (>95.00%) was purchased from the Sigma-Aldrich Corporation (Bellefonte, PA, USA). Methanol (MeOH, HPLC grade) and acetonitrile (ACN, HPLC grade) were purchased from Merck Chemicals (Darmstadt, Germany). Glass sample bottle and polyethersulfone (0.22 μm) syringe filters were purchased from the Waters Corporation (Milford, MA, USA). Ultrapure water (UPW, 18.2 MΩ·cm, TOC < 1 μg/L) was prepared using an Elga Purelab Ultra Analytic system (Bucks, UK). The water hyacinth was obtained from Guangzhou, China. Hydrochloric acid (GR), sodium hydroxide (GR), and calcium chloride (GR) were purchased from Sinopharm Chemical Reagent Co., Ltd. (Shanghai, China). The other reagents were purchased from Kemio Chemical Reagent Co., Ltd. (Tianjin, China) and were all analytically pure.

### 3.2. Preparation of Biochar

First, the roots of the water hyacinth were removed and rinsed three times with UPW, then dried in an oven at 80 °C. It was then pulverized into a powder by a high-speed grinder and sieved through a 300-mesh sieve. For the preparation of the WHBC, the corundum crucible containing water hyacinth powder was first placed into a tubular electric furnace for pyrolysis. The heating rate of the electric furnace was 10 °C/min. The pyrolysis temperature and duration were 400 °C and 120 min, respectively. It should be noted that the pyrolysis process was always under nitrogen protection. WHBC was obtained after pyrolysis treatment and cooling to room temperature.

The raw biochar was modified by mixing CaCl_2_ (2.5 g) and water hyacinth powder (5 g) in 200 mL UPW (the selection of biochar modification ratios see Figure S1), and the mixed solution was ultrasonic water bath treatment for 30 min in an ultrasonic machine. Subsequently, the mixed solution was magnetically stirred at 60 °C for 720 min at a constant temperature by a magnetic agitator. The solid phase in the mixture was separated out and dried (60 °C, 720 min). Finally, the samples obtained in the previous step were heated to 400 °C in a tube furnace at a heating rate of 10 °C/min (the selection of biochar pyrolysis temperatures, see Figure S2), and the modified biochar was obtained after 120 min of pyrolysis treatment under nitrogen protection. The calcium modified WHBC was named WHCBC. 

### 3.3. Characterization

A scanning electron microscope (SEM) equipped with an X-ray energy dispersive spectrometer (EDS) (Sigma 500, Carl Zeiss, Jena, Germany) was used to observe the pore structure of WHCBC, and the composition and content of biochar elements were determined. The specific surface area and porosity of biochar were measured by a surface area analyzer (ASAP 2460, Micromeritics, Norcross, GA, USA). The Fourier transform infrared (FTIR) spectroscopy (Nicolet iS5, Thermo Fisher, Waltham, MA, USA) of biochar was detected using the potassium bromide (KBr) tableting method, and the scanning wave number was in the range of 400–4000 cm^–1^. X-ray diffraction (XRD) patterns were collected using an X-ray diffractometer (Smart Lab 9, Rigaku Corporation, Japan) to determine the crystalline structure formed on biochar. The zero-point charges (pH_iep_) of each biochar were measured by titration using zetasizer analyzer (Zetasizer Nano ZSE, Malvern Panalytical, Malvern, UK). The concentration of metal ions was detected via an inductively coupled plasma–mass spectrometer (ICP-MS 7800, Agilent, Santa Clara, CA, USA).

### 3.4. Adsorption Experiments

The batch experiments (240 min) were carried out under different conditions to investigate the adsorption capacity of WHCBC. Firstly, 0.01, 0.03, 0.05, 0.075, 0.1, and 0.125 g of WHCBC were added into 5 mg/L TE water solution to determine the optimal biochar adsorbent amount. Secondly, WHCBC was added into TE solutions with an initial concentration range of 1 mg/L to 20 mg/L to explore the effect of TE concentration on the adsorption capacity of biochar. Thirdly, the effects of pH (4–10) and temperature (20–45 °C) of TE solution (5 mg/L) on the adsorption of WHCBC were also investigated. The adsorption kinetics of WHCBC with initial TE concentrations of 5 mg/L was investigated, and the adsorption isotherms of WHCBC at solution temperatures of 25 °C, 35 °C, and 45 °C at TE of 1–20 mg/L were also studied, respectively. The effect of coexisting substances on the adsorption of TE (5 mg/L) by WHCBC was studied using the coexisting substances (Cu^2+^, Ca^2+^, Cr^6+^, K^+^, Mg^2+^, Pb^2+^, and HA) at concentrations of 5–20 mg/L. The safety of WHCBC was also investigated in a metal leaching experiment at a solution pH of 3–11. Finally, the recycling performance of the preparation of biochar was explored and determined, and the main steps of its regeneration experiment are as follows. The adsorbent was recovered by suction filtration after each adsorption experiment, and WHCBC was desorbed with 0.2 mol/L HCl. The desorption process was carried out in a thermostatic oscillator while stirring at 175 r/min for 360 min throughout. Thereafter, the biochar was washed with deionized water and dried in an oven at 60 °C for subsequent cyclic adsorption experiments.

All experiments were performed in a thermostatic oscillator (oscillator speed of 175 r/min) with a solution volume of 100 mL. The pH of the TE solution was adjusted with 0.1 mol/L HCl and NaOH in adsorption experiments. The samples were centrifuged (5000 r/min) and filtered (0.22 μm filter membrane) and then detected by ACQUITY^TM^ UPLC system coupled with a triple quadrupole mass spectrometer (UPLC–ESI–MS/MS, Waters Corporation, Milford, MA, USA). The instrument detection conditions are shown in the Supplementary Materials, Text S1. All experiments were conducted in triplicate, and the average values and standard deviations were reported. In addition, statistical analyses were performed using SPSS software (version 26.0), and the results were considered significant at *p* < 0.05. In this study, the adsorption capacity of WHCBC was calculated as follows:

(1) $$q_{e}=\frac{\left(C_{0}-C_{e}\right)V}{m}$$

where *C*_0_ and *C*_e_ are the concentrations of TE in solution at the initial and adsorption equilibrium, mg/L; *V* is the volume of TE solution, L; and *m* is the dosage of WHCBC, g.

## 4. Conclusions

This work demonstrated that low-cost biochar sorbents prepared by CaCl_2_-modified water hyacinth can effectively remove TE from water. The optimal adsorption performance of WHCBC under the influence of various factors was examined by analyzing the parameters such as adsorbent dosage, initial TE concentration, solution pH, and solution temperature. The adsorption of TE from water by WHCBC followed well with the pseudo-second-order kinetics and the Langmuir isotherm model with a maximum adsorption performance of 40.5 mg/g, indicating that the adsorption was mainly monolayer chemical adsorption. Liquid film diffusion and external mass transfer played an important role in controlling the rate of adsorption. The main adsorption mechanisms of TE on WHCBC included hydrogen bond interactions, π–π interactions, and surface complexation. In addition, WHCBC showed excellent adsorption capacity for TE in the presence of coexisting substances. The desorption and reusability experiment indicated that WHCBC biochar had the potential to be a reusable adsorbent for TE removal. Thus, WHCBC can be used as an efficient adsorbent for TE removal from water.

## Figures and Tables

**Table 1:** Specific surface area and pore parameters of the biochars.

Biochar	Specific Surface Area (m^2^/g)	Total Pore Volume (cm^3^/g)	Average Pore Diameter (nm)
WHBC	3.30	0.00430	5.22
WHCBC	5.29	0.00812	6.63

**Table 2:** Adsorption kinetic parameters for TE on the biochars.

Model	Parameter	WHBC	WHCBC
Experimental adsorption capacity	*q*_m_ (mg/g)	3.21	8.01
Pseudo-first-order model	*q*_m_ (mg/g)	2.95	6.98
	*K*_1_ (min^–1^)	0.0575	0.586
	*R* ^2^	0.807	0.805
Pseudo-second-order model	*q*_m_ (mg/g)	3.26	7.45
	*K*_2_ (g/(mg·min))	0.0239	0.105
	*R* ^2^	0.926	0.911
Elovich model	*α* (g/(mg·min)	0.740	261
	*β* (g/mg)	1.72	1.38
	*R* ^2^	0.992	0.989
Intra-particle diffusion model	*k*_id1_ (g/(mg·min^1/2^)	0.471	0.554
	*c*_1_ (mg/g)	0.0300	3.97
	*R*_1_^2^	0.985	0.952
	*k*_id2_ (g/(mg·min^1/2^)	0.153	0.140
	*c*_2_ (mg/g)	1.33	6.38
	*R*_2_^2^	0.919	0.932
	*k*_id3_ (g/(mg·min^1/2^)	0.0253	0.0300
	*c*_3_ (mg/g)	2.82	7.54
	*R*_3_^2^	1.00	0.997
Liquid film diffusion model	*K*_fd_	0.0215	0.0262
	*R* ^2^	1.00	1.00

**Table 3:** Adsorption isotherm model parameters for TE on WHCBC.

Model	Parameter	Temperature (°C)		
		25	35	45
Langmuir isotherm	*q*_m_ (mg/g)	24.0	32.2	40.5
	*K*_L_ (L/mg)	0.618	0.327	0.304
	*R* ^2^	0.989	0.980	0.981
Freundlich isotherm	*K*_F_ (mg/g(L/mg)^1/n^)	10.1	6.38	9.94
	*n*	2.93	1.43	1.76
	*R* ^2^	0.954	0.949	0.995
Sips isotherm	*q*_m_ (mg/g)	26.0	26.9	37.54
	*K*_s_ (L/mg)	0.492	0.510	0.385
	*m*	0.937	1.13	0.839
	*R* ^2^	0.989	0.980	0.985
Temkin isotherm	*K_t_* (L/mg)	12.2	7.71	17.4
	*B*	663	556	507
	*R* ^2^	0.942	0.937	0.973

## Data Availability

Data are contained within the article or the Supplementary Materials.

## Supplementary Materials

The following supporting information can be downloaded at: https://www.mdpi.com/article/10.3390/molecules28083478/s1, Text S1: Parameters of UPLC and MS for analyze of TE; Text S2: Adsorption kinetic model equation; Text S3: Adsorption isotherm model equation; Figure S1: Adsorption capacity of WHCBC with different modification ratios; Figure S2: Adsorption capacity of WHCBC with different pyrolysis temperatures; Figure S3: Adsorption–desorption and pore size distribution of the two biochars: (a) surface area comparison between WHBC and WHCBC; (b) pore size comparison between WHBC and WHCBC; Figure S4: Zeta potential of WHCBC at different solution pH conditions; Figure S5: The leaching concentrations of Ca of WHCBC at different solution pH conditions.
