# Supplementary material for: Efficient Adsorption of Tebuconazole in Aqueous Solution by Calcium Modified Water Hyacinth-Based Biochar: Adsorption Kinetics, Mechanism, and Feasibility
Source: Molecules. 2023 Apr 14;28(8):3478. doi: 10.3390/molecules28083478 (PMC10145345; doi:10.3390/molecules28083478)
Supplement: Supplementary file 1 [file molecules-28-03478-s001.zip › molecules-2331535-supplementary.pdf]

Supplementary Materials

Efficient adsorption of tebuconazole in aqueous solution by calcium modified water hyacinth–based biochar: adsorption kinetics, mechanism, and feasibility

Yucan Liu[1], Zhonglu Gao[1], Xianguo Ji[1], Ying Wang[1], Yan Zhang[1], Hongwei Sun[2*], Wei Li[3], Lide Wang[4], Jinming Duan[5*]

[1]*School of Civil Engineering, Yantai University, Yantai 264005, China*

[2]*School of Environmental and Materials Engineering, Yantai University, Yantai 264005, China*

[3]*Key Laboratory of Northwest Water Resources, Environment and Ecology, Ministry of Education, Xi'an University of Architecture and Technology, Xi'an 710055, China*

[4]*Ningxia Branch of China Design Group Co., ltd., Yinchuan 750001, China*

[5]*Centre for Water Management and Reuse, University of South Australia, Mawson Lakes Campus, Adelaide, SA 5095, Australia*

Corresponding author:

E–mail: sunhw@ytu.edu.cn (Hongwei Sun); phone: +86–15666905528

E–mail: jinming.duan@unisa.edu.au (Jinming Duan); phone: +61–0422746379

TABLE OF CONTENTS

Text S1 Parameters of UPLC and MS for analyze of TE 3

Text S2 Adsorption kinetic model equation 4

Text S3 Adsorption isotherm model equation 5

Figure S1 Adsorption capacity of WHCBC with different modification ratios. 6

Figure S2 Adsorption capacity of WHCBC with different pyrolysis temperature. 7

Figure S3 Adsorption–desorption and pore size distribution of the two biochars: (a) Surface area comparison between WHBC and WHCBC; (b) pore size comparison between WHBC and WHCBC. 8

Figure S4 Zeta potential of WHCBC at different solution pH conditions. 9

Figure S5 The leaching concentrations of Ca of WHCBC at different solution pH conditions (*t* = 240min). 10

Text S1 Parameters of UPLC and MS for analyze of TE

UPLC–ESI–MS/MS was used as the detection instrument; Masslynx 4.1 was used for real-time instrument control, data acquisition, and processing. ACQUITY^{TM} UPLC BEH C18 column (2.1 mm×50 mm×1.7 μm) was used for chromatographic separation. The binary mobile phase was composed of methanol (A) and ultrapure water (B), and the flow rate was 0.2 mL/min. The injection syringe was rinsed automatically after each injection to eliminate interference between injections. Each rinse cycle used 800 μL of weak wash solvent (UPW/MeOH, 90:10, v:v) and 400 μL of strong wash solvent (UPW/MeOH, 10:90, v:v). The elution gradient of UPLC was as follow: 0–1 min, 10% A; 1–5.5 min, 10%–70% A; 5.5–6.5 min, 70%–100% A; 6.5–7 min, 100% A; 7.0–7.1 min, 100%–10% A; 7.1–10 min, 10% A. A total acquisition time of one run analysis was 10 min. The column temperature was maintained at 35 ºC and the injection volume was 10 μL.

Full scan data were acquired from *m*/*z* 50 to 350 at an acquisition rate of 100 ms/scan in both positive electrospray ionization (ESI+) mode and negative electrospray ionization (ESI+) mode. In order to improve the accuracy of the detection, *m*/*z* 308 was used for the quantitative analysis of TE in the selected ion monitoring (SIM, ESI+). The source temperature and desolvation temperature of electrospray ionization-mass spectrometry (ESI-MS) were 110 ºC and 350 ºC, respectively. The capillary voltage and cone voltage were 3 kV and 34 V, respectively. The desolvation gas and cone gas were nitrogen (99.999%) with flow rates of 500 L/h and 50 L/h, respectively.

Text S2 Adsorption kinetic model equation

To quantitatively explain the kinetic process of TE removal by WHCBC, the data were fitted and analyzed by five models, including the pseudo–first–order kinetic model (Eq. 1), pseudo–second–order kinetic model (Eq. 2), Elovich model (Eq. 3), intraparticle diffusion model (Eq. 4), and liquid film diffusion model (Eq. 5):

$$q_t = q_e\,(1 - e^{-K_1 t}) \tag{1}$$

$$q_t = \frac{q_e^2 K_2 t}{1 + q_e K_2 t} \tag{2}$$

$$q_t = \frac{1}{\beta}\ln\,(1 + \alpha\beta t) \tag{3}$$

$$q_t = k_{id} t^{0.5} + c \tag{4}$$

$$ln\,(1 - F) = -k_{fd} t \tag{5}$$

where, q_e (mg/g) and q_t (mg/g) are the adsorption capacity of WHCBC at adsorption equilibrium and adsorption time *t*, respectively; the rate constants of pseudo–first–order and pseudo–second–order kinetics are represented by K_1 (min^{-1}) and K_2 (min^{-1}); α is the initial adsorption rate constant, and β is the desorption rate constant; k_{id} is the intra-particle diffusion rate constant (mg/(g·h$^{1/2}$)), and *C* is the intercept related to the thickness of the boundary layer, that is, the larger the intercept, the greater the contribution of the surface sorption in the rate controlling; k_{fd} represents the adsorption rate constant, and the value of *F* is equal to the ratio of q_t to q_e.

Text S3 Adsorption isotherm model equation

The Langmuir model assumes that the adsorbent surface has uniform adsorption sites and is monolayer adsorption, expressed as:

$$q_e = \frac{q_m K_L C_e}{1 + K_L C_e} \tag{6}$$

The Freundlich model indicates multilayer adsorption on the surface of the heterogeneous adsorbent, denoted as:

$$q_e = K_F C_e^{1/n} \tag{7}$$

The Sips model combines the adsorption behavior of Langmuir and Freundlich model, and establishes the mixed adsorption mechanism, expressed as:

$$q_e = \frac{q_m (K_s c_e)^m}{1 + (K_s c_e)^m} \tag{8}$$

The Temkin isotherm model explains the effect of the adsorbent-adsorbent interaction during the adsorption process, and assumes that the surface binding will reduce the adsorption heat of the adsorbate, denoted as:

$$q_e = \frac{RTlnK_t C_e}{B} \tag{9}$$

where, q_{max} (mg/g) represents the maximum adsorption capacity of WHCBC; K_L and K_F are Langmuir and Freundlich isotherm constants respectively, n is Freundlich intensity parameters; K_S and m are the Sips heterogeneity divisors; The general gas constant is expressed by R, 8.314 J/(mol·K); T denotes the absolute temperature, °C; K_t is equilibrium binding constant, L/g; and B is Temkin constant.

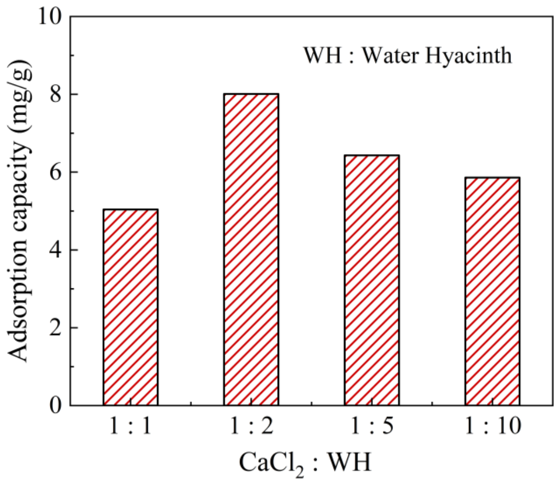

Figure S1 Adsorption capacity of WHCBC with different modification ratios.

Note: the added mass of WH in the pre–experiment is 5 g.

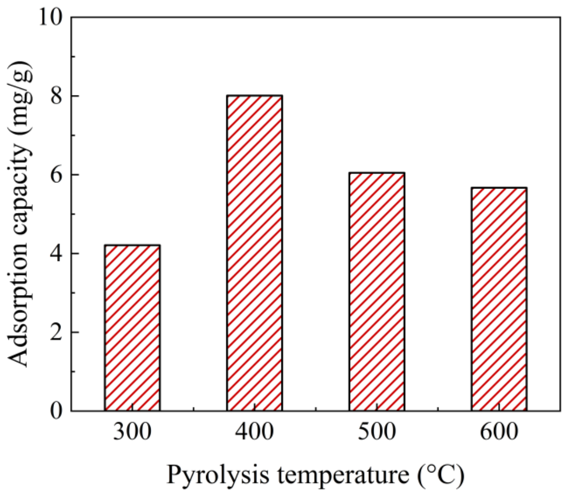

Figure S2 Adsorption capacity of WHCBC with different pyrolysis temperature.

Note: The pyrolysis temperature of 400 °C is the best temperature obtained by investigating the adsorption capacity of biochar in the pre–experiment.

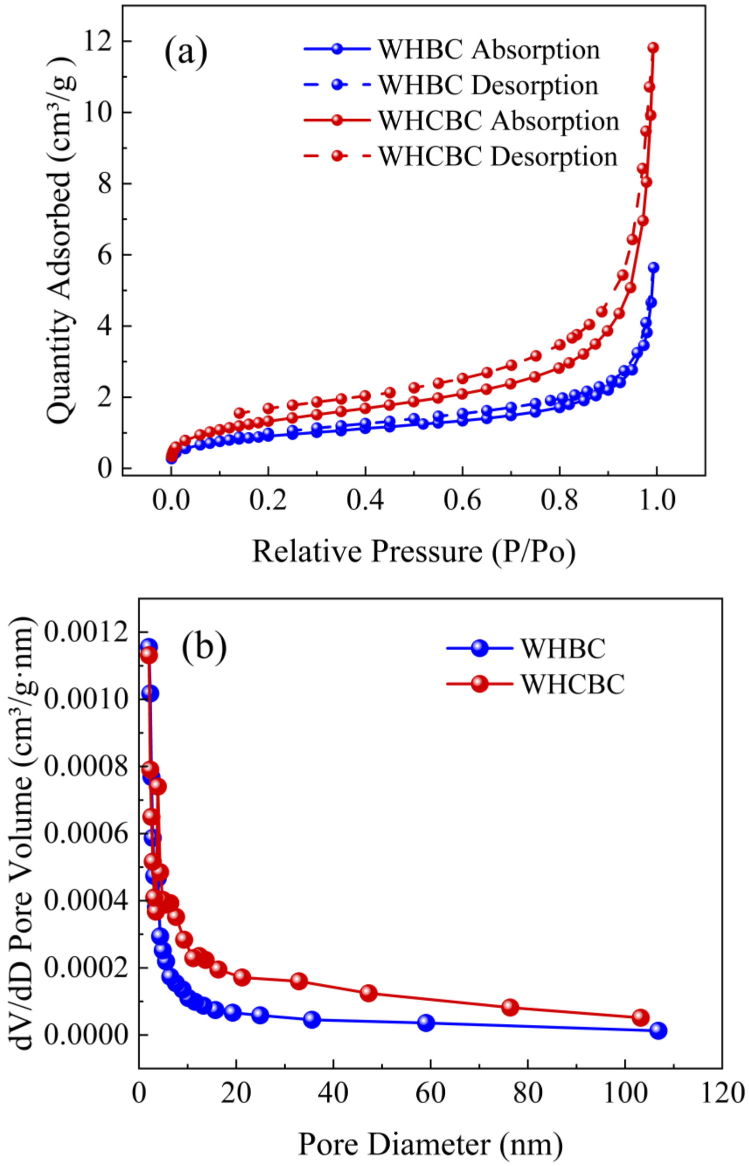

Figure S3 Adsorption–desorption and pore size distribution of the two biochars: (a) Surface area comparison between WHBC and WHCBC; (b) pore size comparison between WHBC and WHCBC.

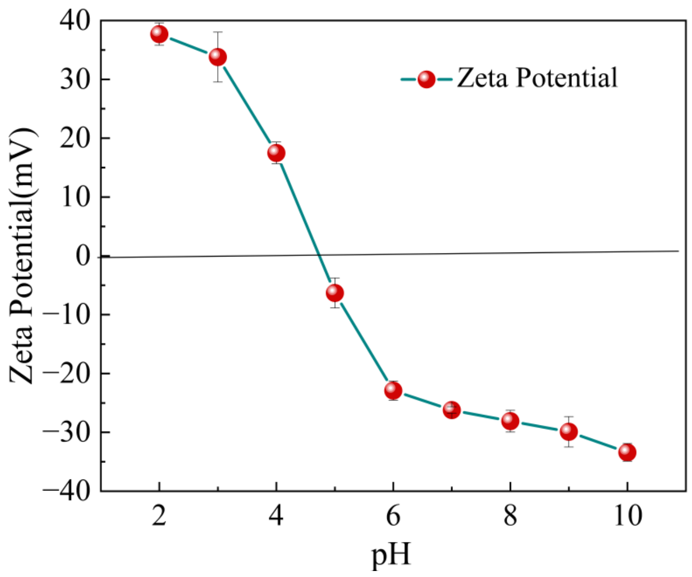

Figure S4 Zeta potential of WHCBC at different solution pH conditions.

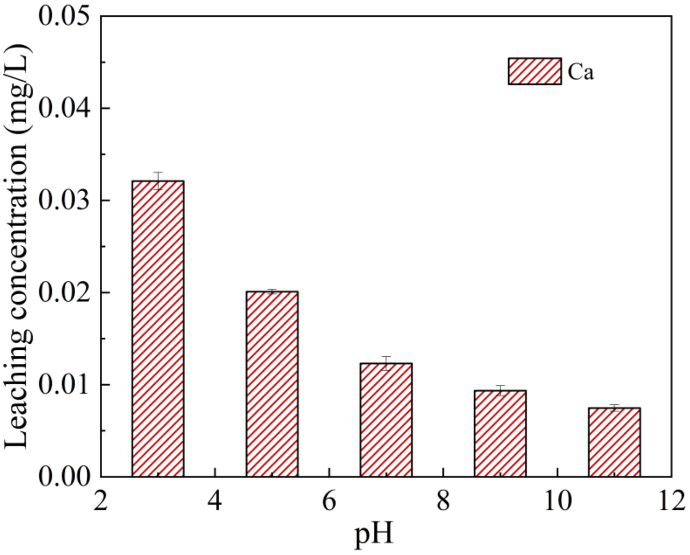

Figure S5 The leaching concentrations of Ca of WHCBC at different solution pH conditions (t = 240min).
